# The gender distribution of psychopathological disorders at a child and adolescent psychiatry department: About 577 cases

**DOI:** 10.1192/j.eurpsy.2021.572

**Published:** 2021-08-13

**Authors:** A. Ben Othman, H. Slama, O. Souid, I. Soualmia, A. Oumaya

**Affiliations:** 1 Child And Adolescent Psychiatry, Military Hospital of Instruction of Tunis, Tunis, Tunisia; 2 Psychiatry, Military Hospital of Instruction of Tunis, Tunis, Tunisia

**Keywords:** psychopathology, Gender

## Abstract

**Introduction:**

The evaluation of the distribution of pathologies according to gender in child and adolescent psychiatry remains an interesting question both for epidemiological and etiopathogenic research.

**Objectives:**

The objective of our work was to study the influence of gender on the psychopathological expression of disorders in children and adolescents.

**Methods:**

A retrospective study was conducted at the outpatient child psychiatry department at the Tahar Sfar University Hospital in Mahdia, Tunisia, among patients who consulted for the first time during the period from January 2015 to December 2017. To carry out this work, we used a pre-established form evaluating the sociodemographic and clinical information. We opted for grouping diagnoses according to axis I of the DSM IV- TR.

**Results:**

We collected 577 cases: 231 girls, and 346 boys. The analysis of the results showed a male predominance (60% of our sample were boys). A significant difference in diagnosis relating to gender was found (p = 0.02). In fact, boys were more likely to have pervasive developmental disorders, mental retardation and externalizing disorders, while girls were more likely to develop sleep and eating disorders and internalizing disorders such as mood disorders.

**Conclusions:**

Demonstrating the existence of a significant gender distribution of pathologies in adolescent and child psychiatry is certainly of real importance in the context of epidemiological, clinical and etiopathogenic research. However, it would be more efficient to further investigate other epidemiological and socio-cultural data in order to better specify the involvement of each of these factors in the emergence of psychopathology.

